# Developing a Semantically Based Query Recommendation for an Electronic Medical Record Search Engine: Query Log Analysis and Design Implications

**DOI:** 10.2196/45376

**Published:** 2023-09-15

**Authors:** Danny T Y Wu, David Hanauer, Paul Murdock, V G Vinod Vydiswaran, Qiaozhu Mei, Kai Zheng

**Affiliations:** 1 Department of Biomedical Informatics University of Cincinnati College of Medicine Cincinnati, OH United States; 2 School of Information University of Michigan Ann Arbor, MI United States; 3 Department of Learning Health Sciences University of Michigan Ann Arbor, MI United States; 4 Burnett School of Medicine Texas Christian University Fort Worth, TX United States; 5 Department of Biomedical Informatics University of Cincinnati Cincinnati, OH United States; 6 Department of Informatics University of California Irvine, CA United States

**Keywords:** electronic health records, information retrieval, user-centered evaluation, query recommendation, query log analysis, clinical research informatics

## Abstract

**Background:**

An effective and scalable information retrieval (IR) system plays a crucial role in enabling clinicians and researchers to harness the valuable information present in electronic health records. In a previous study, we developed a prototype medical IR system, which incorporated a semantically based query recommendation (SBQR) feature. The system was evaluated empirically and demonstrated high perceived performance by end users. To delve deeper into the factors contributing to this perceived performance, we conducted a follow-up study using query log analysis.

**Objective:**

One of the primary challenges faced in IR is that users often have limited knowledge regarding their specific information needs. Consequently, an IR system, particularly its user interface, needs to be thoughtfully designed to assist users through the iterative process of refining their queries as they encounter relevant documents during their search. To address these challenges, we incorporated “query recommendation” into our Electronic Medical Record Search Engine (EMERSE), drawing inspiration from the success of similar features in modern IR systems for general purposes.

**Methods:**

The query log data analyzed in this study were collected during our previous experimental study, where we developed EMERSE with the SBQR feature. We implemented a logging mechanism to capture user query behaviors and the output of the IR system (retrieved documents). In this analysis, we compared the initial query entered by users with the query formulated with the assistance of the SBQR. By examining the results of this comparison, we could examine whether the use of SBQR helped in constructing improved queries that differed from the original ones.

**Results:**

Our findings revealed that the first query entered without SBQR and the final query with SBQR assistance were highly similar (Jaccard similarity coefficient=0.77). This suggests that the perceived positive performance of the system was primarily attributed to the automatic query expansion facilitated by the SBQR rather than users manually manipulating their queries. In addition, through entropy analysis, we observed that search results converged in scenarios of moderate difficulty, and the degree of convergence correlated strongly with the perceived system performance.

**Conclusions:**

The study demonstrated the potential contribution of the SBQR in shaping participants' positive perceptions of system performance, contingent upon the difficulty of the search scenario. Medical IR systems should therefore consider incorporating an SBQR as a user-controlled option or a semiautomated feature. Future work entails redesigning the experiment in a more controlled manner and conducting multisite studies to demonstrate the effectiveness of EMERSE with SBQR for patient cohort identification. By further exploring and validating these findings, we can enhance the usability and functionality of medical IR systems in real-world settings.

## Introduction

### Background and Significance

Clinical documentation is a central component of patient care, fundamental to clinicians’ ability to review patients’ medical history, makes sense of current medical problems, and determines optimal treatment and care plans [1]. Clinical documentation is usually accomplished through the use of electronic health record (EHR) systems, which collect a massive amount of detailed computerized patient data [2]. In addition to direct use in clinical care, EHR data are valuable to research, administration, billing, and medical education. Among these secondary uses, supporting clinical and translational research has been of great interest due to EHRs’ potential to reduce the cost of research data collection, improve the efficiency of study conduction, and achieve higher study generalizability [3,4].

Despite the great potential, the secondary uses of EHR data to support clinical and translational research can be difficult. One major difficulty is that a significant portion of EHRs is recorded in free-text form such as progress notes and discharge summaries, making the information locked in sentences and not easily available. These free-text data are highly expressive, flexible, and compatible with the workflow [5] and can provide great insight into patients’ medical conditions. To use this information, it is common to use natural language processing techniques to extract information and identify patient cohorts [6,7]. However, natural language processing techniques are sometimes suboptimal due to a large amount of required training data, low usability of system output, and the performance tuned for local context [8]. On the other hand, information retrieval (IR) systems can help users pinpoint information of interest and have been shown to be a viable, scalable, and user-friendly solution to leverage medical information recorded in free-text form.

An electronic medical record search engine (EMERSE) was developed at the University of Michigan [9]. EMERSE has been used to index large-scale routine medical documents for the past 18 years [10] and is now being deployed at other academic medical centers. To enhance the performance of EMERSE, a semantically based query recommendation (SBQR) feature was developed in its early version and evaluated to help users expand or substitute original search queries and further improve the accuracy and relevancy of their search results. Our previous evaluation study showed that the SBQR exhibits high performance when evaluated by prospective end users [11]. This follow-up study conducted further analysis to demonstrate how SBRQ may contribute the positive feedback from the end users.

### Literature Review

IR consists of a set of algorithms and techniques for effectively storing free-text documents and retrieving relevant documents to meet users’ information needs. A common challenge of IR is that users may have limited knowledge of what they are searching for. As Bates [12] pointed out, users seek information in an evolving manner to deal with uncertainties that originate from a complex search environment. An IR system needs to be carefully designed, especially its user interface, to help users work through this “berry-picking” process where users’ information needs and queries change when relevant documents are found in the search process [12]. These uncertainties are especially prominent in the medical domain. That is, clinicians and clinical researchers often have difficulties when formulating effective queries even with medical training and domain knowledge. It is even more challenging when searching for information in EHRs due to the complex nature of clinical documentation (eg, the use of interchangeable terms and nonstandard acronyms and abbreviations) [13,14].

One solution to addressing these issues is to use “query recommendation,” which has been an integral component in modern IR systems such as Google and Microsoft Bing. Query recommendation improves IR performance by suggesting alternative queries, which are derived from query log analysis and mining [15,16] so that the IR system can retrieve more relevant documents and better match user needs. Query recommendations based on the content (plain text) of the documents can already boost the performance of a medical IR system by considering abbreviations, acronyms, and synonyms in EHRs. For example, the term “pat” can be a shortening of the word “patient” in certain situations but may also refer to “paroxysmal atrial tachycardia” in the medical context. On the other hand, query recommendations based on the medical concepts and semantics in the documents can be very powerful. For example, clinicians who search “car accident” in EHRs would appreciate the search results including terms such as “vehicle accident” or “motor accident” because of their similar meanings. While the SBQR has been studied and applied to IR in biomedical literature, images, and medical records [17-20], few studies have investigated end users’ perceptions on its performance. Our previous study addressed this gap by designing a prototype system with the SBQR [21] and evaluated the system with 33 prospective end users (study participants) including clinicians, clinical researchers, and health care administrators [11]. Each user was given 5 scenarios representing realistic information needs to search EHR notes. Each scenario was carefully designed with a difficulty level estimated by the research team (low, medium, or high). The results of our previous study showed that these participants had positive perceptions toward the SBQR, suggesting beneficial effects when implementing an SBQR in medical IR systems. Most of the participants would like to use the SBQR to assist with their day-to-day EHR search tasks. This study further analyzed the query logs and aimed to provide empirical evidence for the positive perception of the system performance and examined the role of the SBQR.

## Methods

### Electronic Medical Record Search Engine

**Figure 1:**
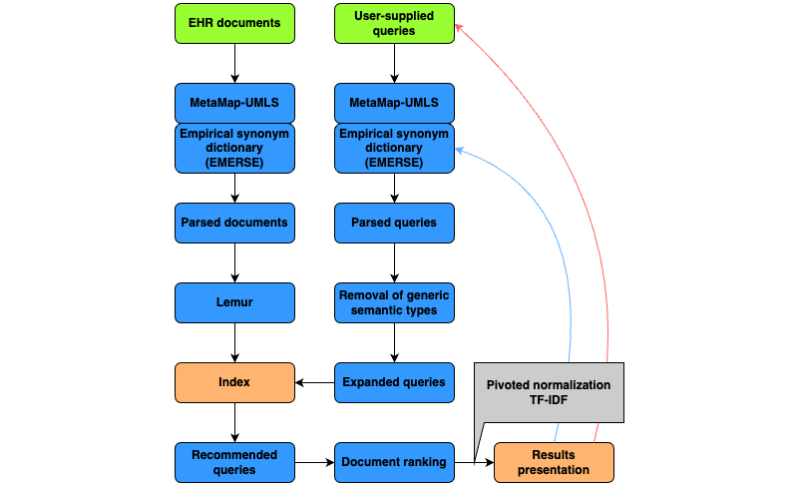
Workflow of semantically based query recommendation feature [21]. EHR: electronic health record; EMERSE: Electronic Medical Record Search Engine; IDF: inverse document frequency; TF: term frequency; UMLS: unified medical language system.

The query log data analyzed in this paper were collected from our previous experimental study on EMERSE with SBQR [11]. As illustrated in Figure 1, the SBQR mapped user-supplied queries to corresponding medical concepts using MetaMap (National Library of Medicine) [22]. These medical concepts were then expanded to include synonyms based on two synonym sets: (1) a predefined set by the Unified Medical Language System and (2) an empirical collection from EMERSE based on historical searching records [9]. The SBQR-expanded queries were further matched to the indexes of the corpus containing about 100,000 medical documents. These indexes were constructed following the same mapping process. With the assistance of the SBQR, the prototype system was expected to recall more relevant documents compared to it without the SBQR. For example, with the SBQR, users can obtain documents containing both “hearing loss” and “difficulty of hearing” when searching with either term.

### User Information-Seeking Behaviors

**Figure 2:**
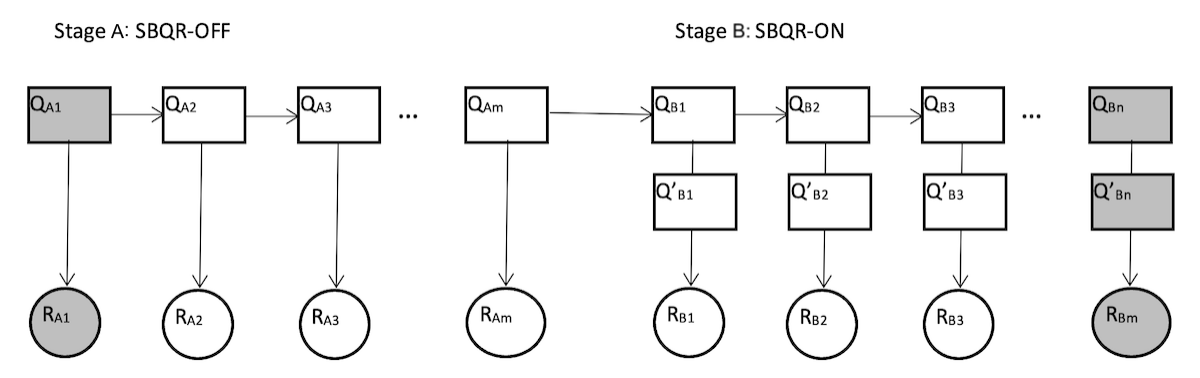
A sample and abstracted search process. Q_A1_ is the first query that a user enters when the SBQR is turned off, resulting in the retrieved documents R_A1_. Users can try as many queries as they would like to and then turn the SBQR on manually. Q_Bn_ is the last user-supplied query when the SBQR is turned on, which is expanded to Q’_Bn_ by the SBQR, resulting in the retrieved documents R_Bm_. SBQR: semantically based query recommendation.

A logging mechanism was developed to keep track of the user query behaviors and the IR system output (retrieved documents). Specifically, the logs included the original user-supplied queries, the parsed medical concepts, the expanded terms when the SBQR was turned on, the top 30 retrieved documents, and the timestamps of each behavior. Five predefined scenarios with various difficulty levels (low, medium, or high), which were predefined by the research team, were given to all 33 participants in the user experiment (Table 1). The order of the scenarios matches the order given to each participant. In each scenario, the participants were told to turn the SBQR off and formulate as many queries as needed until they retrieved satisfactory results. The participants were then asked to turn the SBQR on and conduct another round of searching until they reached another set of satisfactory results. Figure 2 illustrates a general search process of a user in a scenario, highlighting the selected queries used for the analysis. To determine whether the use of the SBQR helped in constructing improved queries, the initial query that a user entered (Q_A1_) was compared with the query achieved with the assistance of the SBQR (Q_Bn_). The study did not compare the last query that a user entered (Q_Am_) and the last query assisted by the SBQR (Q_Bn_) because users can freely turn SBQR on and off multiple times during the experiment to mimic real search behaviors. Such freedom, however, made the determination of Q_Am_ very difficult.

**Table 1:** Summary of query activities.

Scenario	Estimate difficulty	Average number of queries per user	Average % of query with SBQR^a^	Average number of terms per user query	Average number of characters per user query
1^b^	Medium	9.33	36.1	2.69	32.26
2^c^	Low	8.24	45.2	2.56	27.12
3^d^	Medium	13.45	43.2	3.54	36.21
4^e^	High	8.24	46.5	3.28	44.28
5^f^	Medium	6.97	45.5	3.02	34.03

^a^SBQR: semantically based query recommendation.

^b^Scenario 1: You are doing a research project in which you want to identify people who have had a concussive episode after being in a car accident.

^c^Scenario 2: You are interested in identifying patients who have the noninvasive form of breast cancer known as intraductal carcinoma (DCIS).

^d^Scenario 3: Please try to identify patients who are smokers who have also been diagnosed with posttraumatic stress disorder.

^e^Scenario 4: You are interested in how many patients are taking herbal supplements for the purposes of weight loss, high.

^f^Scenario 5: Someone has asked you to determine if we have many patients diagnosed with mono who had an enlarged spleen.

### Log Analysis

The query log analysis contained 3 parts. First, the participants’ query activities were summarized by the number of queries per user, the number of terms per query per user, and the percentage of queries that the participants submitted when the SBQR was turned on. Second, a similarity analysis was conducted to compare the initial query that a user entered (Q_A1_) and the last SBQR-assisted query (Q_Bn_) of each user in each scenario. The difference between Q_A1_ and Q_Bn_ was measured by the degree of term overlapping. That is, terms contained in Q_A1_ were extracted and converted to their lower-case form and merged to construct a term vector (V_A1_) in each user scenario. The term vector of Q_Bn_ (V_Bn_) was formed in the same way. The similarity between Q_A1_ and Q_Bn_ was measured by the Jaccard similarity coefficient of the 2-term vectors. The coefficient was defined as the ratio of intersection between the terms of these 2 vectors over the union of them (equation 1). It is hypothesized that the queries were not significantly different. This hypothesis is based on the tendency that users rely more on an IR system to automatically manipulate their queries than on their own to modify the queries. In other words, if the queries are shown to be very similar (eg, Jaccard similarity coefficient ≥0.7), users would not make much effort to change the queries. Therefore, any perceived positive system performance at the end of each scenario would likely come from the automatic query modifications by the SBQR rather than the manual query changes by the users.

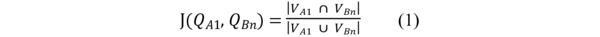



**Figure 3:**
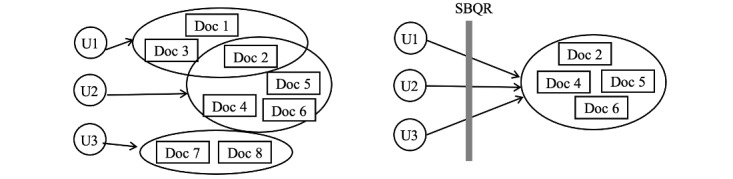
The effectiveness of the SBQR. Participants with the same information needs may use different search strategies (eg, terms), resulting in lower overlap between retrieved documents (left, high uncertainty). However, with the assistance of the SBQR, these users may be “brought together” by the SBQR and therefore retrieved a similar set of documents. SBQR: semantically based query recommendation.

The third part of the query log analysis explored the relationship between the SBQR, the search results, and the perceived system performance through an entropy analysis. As illustrated in Figure 3, it is hypothesized that different participants would return similar results with the help of the SBQR given the semantic overlap of concepts in their queries when compared to the queries of each person without the assistance of the SBQR. Therefore, participants with the same information needs but different search strategies would retrieve a similar (or a converged) set of documents using the SBQR. In other words, the SBQR helped “standardize” the queries across multiple users. This hypothesis was examined by comparing the entropy of the result sets, that is, comparing R_A1_ and R_Bn_ in Figure 2. A result set is defined as the union of the top 10 documents retrieved in each search scenario. The entropy analysis gauged the level of uncertainty in a probability distribution, which is constructed by a set of probability scores from the retrieved documents in the result set. The probability score of a document was calculated using the ratio of the frequency of a document to the sum of the frequency of all the documents in the result set. These probability scores were turned into an entropy score using the formula described in (equation 2). As equation 2 shows, the variable “*x*” represents the probability score of 1 resultant document. If the hypothesis is true, a decreased entropy score would be observed, leading to a negative difference in entropy between R_A1_ and R_Bn_.

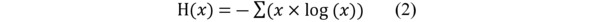



### Statistical Analysis

The difference in Jaccard similarity scores was examined statistically. First, the normality of the scores in each scenario and the homogeneity of the variance among scenarios were tested using the Kolmogorov-Smirnov test for goodness of fit and the Bartlett/Levene test for variance equality, respectively. If the distribution of the Jaccard similarity scores was not normal, a nonparametric test (Kruskal-Wallis) was used to examine group median differences. Query log data were stored and processed in a standalone SQLite database. Two data views were created to capture the first and last query of each user scenario. The procedures to calculate Jaccard similarity and Entropy were implemented in Python (version 2.7; Python Software Foundation) with libraries “pandas,” “numpy,” and “scipy.” The use of the query log data was approved by the University of Michigan Health Sciences and Behavioral Sciences institutional review boards [11].

### Ethical Considerations

#### Ethics Approval

This study received ethics review and approval from the Human Research Protection Program at the University of Michigan (HUM00057979) prior to its commencement. The study protocol, including the data collection procedures and informed consent process, was reviewed to ensure compliance with ethical guidelines for conducting research involving human subjects.

#### Privacy and Confidentiality Protection

To protect the privacy and confidentiality of the participants, several measures were implemented. All collected data, including query logs and retrieved documents, were anonymized or deidentified to ensure that no personally identifiable information could be linked to individual participants. Access to the data was restricted to authorized researchers involved in the study, and the data were securely stored on servers with restricted access. Data transmission was encrypted to prevent unauthorized interception or access.

#### Secondary Analysis of Research Data

The original informed consent (or the institutional review board approval) allowed for such secondary analyses without the need for additional consent. The data used in this study were obtained from a previous study, and the original informed consent process allowed for the analysis of the data for research purposes.

## Results

### Query Activities

The search activities of the 33 participants resulted in 10,451 records with 2098 distinct queries. Table 2 shows an example of a participant’s queries in scenario 2. The description of the scenarios can be seen in Table 1. The sample records in Table 2 show that User 005 submitted a total of 6 queries, with 2 of them being SBQR-expanded queries (the fourth and fifth queries). The user started with a single term query “dcis” without the SBQR and manually modified the query to “non-invasive dcis.” The user ended up with a query consisting of 2 concepts: “dcis” and “breast cancer.”

The search activities of all scenarios are summarized in Table 1. Participants formulated between 6 and 13 queries, with nearly half constructed with the SBQR turned on. Scenario 1 was an exception, of which two-thirds of the queries were submitted when the SBQR was turned off. The queries contained 2 to 4 terms, and their length was between 27 and 44 characters. In the scenario with low difficulty, the participants formulated shorter queries, both in terms of number of terms (2.56) and the average number of characters (27.12). On the other hand, the participants tended to formulate longer queries with more terms (3.28) and characters (44.28) in the scenario with high difficulty.

**Table 2:** A set of queries entered by user 005 (U005) in scenario 2 “You are interested in identifying patients who have the noninvasive form of breast cancer known as DCIS.” All query logs were uploaded in Multimedia Appendix 1.

Log ID	Seq	Query	SBQR^a^	Time stamp
1000006702^b^	1	dcis	Off	09:54:34
1000006712	2	non-invasive dcis	Off	09:55:11
1000006717	3	non-invasive dcis breast cancer	Off	09:55:30
1000006762	4	non-invasive dcis breast cancer	On	09:57:39
1000006776^a^	5	dcis, breast cancer	On	09:59:23
1000006795	6	dcis, breast cancer	Off	10:00:30

^a^SBQR: semantically based query recommendation feature.

^b^Queries in Sequences 1 and 5 were selected for analysis: the first query when the SBQR was turned off (1000006702, Q_A1_) and the last query when the SBQR was turned on (1000006776, Q_Bn_).

### Similarity Analysis

The average Jaccard similarity coefficient of each scenario is reported in Table 3. Overall, the Jaccard similarity coefficient shows an average of 0.77 similarity between the initial query without SBRQ (Q_A1_) and the last query with SBQR (Q_Bn_) in all scenarios. Both queries were entered by the users. The Kolmogorov-Smirnov test and Bartlett/Levene test showed the distribution of the Jaccard coefficient in each scenario was non-normal (*P*=.01) with equal variance (*P*=.15). Then, the Kruskal-Wallis test shows no significant difference between the medians of the coefficient scores (*P*=.26). As shown in Table 3, scenario 1 (first in the order) and scenario 4 (high difficulty) had a lower Jaccard similarity coefficient than the others, while scenario 2 (low difficulty) has the highest Jaccard similarity coefficient. Together with the query analysis in Table 1, participants submitted shorter and similar queries in easier scenarios and longer, varying queries in more challenging ones.

**Table 3:** Summary of query similarities.

Scenario	Estimate difficulty	Average Jaccard coefficient, mean (SD)	Median Jaccard coefficient
1	Medium	0.69 (0.35)	0.8
2	Low	0.86 (0.22)	1.0
3	Medium	0.80 (0.32)	1.0
4	High	0.71 (0.37)	1.0
5	Medium	0.79 (0.33)	1.0

Overall, the results show that participants did not have much change in their queries after the assistance of the SBQR. The high Jaccard similarity coefficient (0.77) between the initial query without SBQR (Q_A1_) and the last query with SBRQ (Q_Bn_) indicates the small change, suggesting that the observed positive perception of end users toward our prototype system likely came from the SBQR, which modified the last query and may help retrieve more relevant documents. Since participants entered similar queries at the end of the search process, variations in queries were not large and likely did not influence the perceived system performance much. Rather, the SBQR changed the search results and affected the perceived system performance. If participants were to formulate different queries, the perceived system performance would have been affected by both the variation in the user-supplied queries and their SBQR-expanded form. In this case, it would be challenging to separate contributing factors and explain the correlation between SBQR use and the perceived system performance.

### Entropy Analysis

The results of the entropy analysis support our second hypothesis. As listed in Table 4, a negative difference in the entropy scores was observed in scenarios 1, 3, and 5 (all with medium difficulty), suggesting that the SBQR helped standardize the queries submitted by different participants. However, a positive gain in entropy scores was observed in scenarios 2 and 4, indicating that the result sets were more variable after the SBQR was turned on. Further analysis shows that differences in entropy scores were highly correlated to participants’ perceived system performance (Pearson correlation coefficient –0.85). This high negative correlation suggests that a converged result set when using the SBQR may lead participants to a higher perceived system performance. It seems that in a high-difficulty scenario, the participants formulated very different queries with distinct medical concepts due to their limited knowledge, preventing the retrieved results from being converging even with the help of the SBQR. On the other hand, in an easier scenario where information needs are clear, the SBQR may introduce “noise” into the result set by adding semantically related but not closely relevant terms. Of note, the SBQR mapped queries with similar medical concepts into similar queries, resulting in similar result sets. The SBQR did not examine whether the user-supplied queries were correct or accurate in each of the scenarios.

**Table 4:** The analysis of the entropy of the top 10 result sets. The Pearson correlation between the entropy difference (%) and the perceived performance was –0.85.

Scenario	Estimate difficulty	Entropy SBQR^a^-Off	Entropy SBQR-On	Entropy difference, n (%)	Perceived performance
1	Medium	4.4384	3.6747	–17.21^b^	4.24
2	Low	3.0688	3.3858	10.33	3.94
3	Medium	3.8411	3.5537	–7.48^a^	4.42
4	High	3.9398	4.0709	3.33	4.09
5	Medium	3.6617	2.9248	–20.12^a^	4.55

^a^SBQR: semantically based query recommendation.

^b^Scenarios 1, 3, and 5 show a negative entropy.

## Discussion

### Principal Findings

Using unstructured clinical data require IR techniques to effectively assist clinicians in finding relevant information in free-text documents. In our previous study, a prototype medical IR system with an SBQR feature was developed and evaluated to show its perceived positive performance with 33 prospective end users. In this study, the query logs were analyzed to generate empirical evidence and potential explanations for the participants’ positive perceptions toward the system. The results showed that participants formulated similar queries with the assistance of the SBQR, suggesting that perceived positive system performance was likely contributed by the SBQR rather than the manual modifications of the queries. Moreover, the participants tended to formulate shorter and more similar queries in an easy scenario and longer and less similar queries in more challenging scenarios.

The results of the entropy analysis suggest that the estimated difficulty level of the scenarios was a contingent factor on participants’ perceived system performance. The SBQR achieved higher performance in scenarios with a medium difficulty as opposed to the extremes (a low or high difficulty). One explanation could be that in the extreme case, the SBQR provided limited help and introduced noise. This finding provides 2 insights into how to incorporate an SBQR in modern medical IR systems, such as EMERSE. First, an SBQR could be designed as a user-controlled option, in which user input is necessary to determine when to turn the feature on or off. Second, an SBQR can be designed as a semiautomated feature, which is activated based on the observed and inferred difficulty of users’ information needs, potentially through real-time analysis of query terms and retrieved documents [23-25].

### Strengths and Limitations

The strength of this study lies in using a combination of objective query logs and self-report survey data to uncover participants’ complicated search behaviors when using the SBQR feature to search EHR notes. The study has several limitations. First, the SBQR was not strictly controlled in the user experiment. The participants alternated between turning the SBQR on and off. While this allowed participants to conduct searches more naturally, it added complexity to the analysis. To mitigate this issue, our analysis focused on the initial query that a user entered when the SBQR was turned off and the last query when the SBQR was turned on. Second, the participants were not asked to provide relevant feedback on the retrieved documents nor was there a gold standard of document relevance for each scenario. Since this study did not collect relevant feedback from the users, standard IR metrics such as normalized discounted cumulative gain cannot be calculated to generate more direct evidence. The document-level relevance feedback was not collected because ranking has almost no meaning in this type of search. The search goal of EMERSE is identifying patient cohorts rather than ranking relevant patients on the top of the pages. We, therefore, conduct the entropy analysis to show a high correlation between the perceived system performance and a high degree of convergence of the retrieved results among participants. Of note, our analysis mainly focused on the top 10 retrieved documents across the 33 participants because each participant may review a varied number of documents in the experiment. Only including the top 10 documents may not be very reflective of the cohort identification process, in which many more patients or documents could be needed. In the same vein, it would be challenging to ascertain that the change in entropy would represent the change in document variety. Next, the difficulty level of each scenario was estimated by the research team. User-indicated difficulty levels can help improve the validity of the entropy analysis and will be considered in our follow-up studies. Finally, this study only analyzed the SBQR in 1 prototype system. The SBQR can be implemented in multiple EHR search engines and used in multiple institutions to examine the effectiveness and generalizability of the SBQR.

Our future work is 2-fold. First, we will learn from this study and redesign the experiment in a more controlled manner to demonstrate the effectiveness of the SBQR in improving the quality of input queries and search results. Second, we will implement SBQR in EMERSE and deploy the feature to the participating sites. We will collect query and usage logs and compare search behaviors across multiple institutions. We will also develop a query standardization and exchange platform to facilitate patient cohort identification across institutions to support large-scale clinical research studies.

### Conclusions

This study analyzed the query logs in a user experiment of a prototype EHR search engine with the SBQR and provided empirical evidence as well as potential explanations for the perceived system performance. The results show that the positive perception was likely attributed to the effectiveness of SBQR and was contingent upon the difficulty level of a particular search scenario. This study confirms that an SBQR has the potential to overcome challenges when retrieving medical documents. Modern medical IR systems, such as EMERSE, should consider the design of an SBQR as a user-controllable option or a semiautomated feature that is triggered when the difficulty of a user’s information needs can be assessed or inferred.

## Appendix

Multimedia Appendix 1 Log data analyzed in the manuscript.

## Abbreviations

EHR: electronic health record
EMERSE: Electronic Medical Record Search Engine
IR: information retrieval
SBQR: semantically based query recommendation

## Data Availability

The query logs analyzed in this study were deidentified and uploaded as Multimedia Appendix 1.
